# The Global Health Security Index and Its Role in Shaping National COVID‑19 Response Capacities: A Scoping Review

**DOI:** 10.5334/aogh.4625

**Published:** 2025-03-14

**Authors:** Danik Iga Prasiska, Kennedy Mensah Osei, Durga Datta Chapagain, Vasuki Rajaguru, Tae Hyun Kim, Sun Joo Kang, Sang Gyu Lee, Suk-Yong Jang, Whiejong Han

**Affiliations:** 1 Global Health Security, Graduate School of Public Health, Yonsei University, Seoul, South Korea; 2 Healthcare Management, Graduate School of Public Health, Yonsei University, Seoul, South Korea; 3 Department of Global Health and Disease Control, Graduate School of Public Health, Yonsei University, Seoul, South Korea; 4 Department of Preventive Medicine, College of Medicine, Yonsei University, Seoul, South Korea

**Keywords:** COVID‑19, Global Health, Index, Response, Security

## Abstract

*Introduction:* Following the introduction of the Global Health Security Index (GHSI), the coronavirus disease 2019 (COVID‑19) pandemic emerged as an unprecedented global health crisis, underscoring the need for robust health security frameworks and preparedness measures. This study conducts a scoping review to analyze the existing literature on the GHSI and assess national COVID‑19 responses across different countries.

*Method:* A comprehensive search of electronic databases (EBSCO, EMBASE, PubMed, Scopus, and Web of Science) was conducted for articles published from 2020 to 2024. Search terms included “Global Health Security Index” and terms related to COVID‑19. The study followed the Preferred Reporting Items for Systematic Reviews and Meta‑analyses for Scoping Reviews (PRISMA‑ScR) guidelines. The Newcastle–Ottawa Scale (NOS), adjusted for cross‑sectional studies, was used for quality assessment.

*Results:* A total of 3,243 studies were identified, of which 20 were finalized for data synthesis. Specific COVID‑19 parameters were analyzed to provide a comprehensive overview of each country’s pandemic response capacity. Among the selected studies, 17 (85%) had a low risk of bias, while 3 (15%) had a medium risk. Countries’ response capacities were categorized into five key parameters: detection, mortality, transmission, fatality, and recovery. Findings revealed significant discrepancies between GHSI scores and actual national responses, with some high‑scoring countries struggling to control the pandemic. This raises concerns about the GHSI’s predictive reliability.

*Conclusion:* The study highlights that the GHSI does not fully capture a country’s capacity to respond effectively to COVID‑19. However, it remains a valuable tool for identifying gaps in pandemic preparedness. To enhance its relevance, the index should integrate a wider range of factors, including political leadership, governance, public health infrastructure, and socio‑cultural elements, which are crucial in managing public health emergencies.

## Introduction

The Global Health Security Index (GHSI) represents a pioneering effort to evaluate and benchmark health security and related capacities across all 195 countries that are States Parties to the International Health Regulations. This comprehensive assessment aims to drive tangible improvements in national health security preparedness while strengthening global readiness to address infectious disease outbreaks. By focusing on measurable enhancements, the GHSI seeks to bolster international capabilities to swiftly detect, respond to, and mitigate the impact of epidemics and pandemics, thereby safeguarding global health and stability in an interconnected world [1].

Shortly after the launch of the GHSI, the coronavirus disease 2019 (COVID‑19) pandemic emerged as an unprecedented global health crisis. Caused by the novel coronavirus severe acute respiratory syndrome coronavirus 2 (SARS‑CoV‑2), COVID‑19 has been described as the most significant health threat of the 21st century. Originating in Wuhan, China, toward the end of 2019, the virus rapidly spread across continents, prompting the World Health Organization to declare it a pandemic in March 2020 [2]. The rapid transmission [3] and severe health consequences [4, 5] underscore the critical importance of robust health security frameworks and preparedness measures, highlighting the urgent need for continuous advancements in global health security strategies to effectively manage future pandemic risks.

The COVID‑19 pandemic has profoundly impacted healthcare systems globally [6–8], reverberating across multiple sectors of the global economy. Frontline healthcare workers have been particularly vulnerable due to their direct exposure to infected patients, placing them at heightened risk of contracting SARS‑CoV‑2. This underscores the necessity of robust infection control measures and adequate personal protective equipment (PPE). Simultaneously, healthcare delivery for the general population has faced significant challenges, exacerbated by surging demand for medical supplies, restrictions on in‑person medical consultations due to infection control protocols, and shortages of essential protective gear. These disruptions have necessitated rapid adaptations in healthcare delivery, emphasizing telemedicine and digital health [9] solutions to maintain continuity of care while minimizing transmission risks. Addressing these complexities requires concerted efforts to strengthen healthcare infrastructure, enhance supply chain resilience, and support frontline healthcare workers in managing ongoing and future health crises [10].

Beyond health consequences, efforts to contain the pandemic led to extensive lockdowns [11] and social distancing [12] measures, which resulted in significant economic losses for individuals, businesses, and governments, with some countries facing the risk of recession [10]. Financial markets have been affected, and disruptions to international supply chains have been widely observed [13].

The COVID‑19 pandemic has served as both a test of health security infrastructures worldwide and a real‑world validation of the Global Health Security Index (GHSI). The exponential rise in infections, mortality rates, and widespread transmission has reinforced findings from the GHSI, revealing that no country was fully prepared for a pandemic of this scale [14, 15]. However, discrepancies have emerged between GHSI scores and the actual responses of some countries, sparking debate and controversy. These inconsistencies highlight the complexities of assessing and predicting pandemic preparedness, emphasizing the need for continuous refinement of global health security frameworks to better mitigate future crises [16].

This scoping review examines the existing scientific literature on the impact of the Global Health Security Index (GHSI) on COVID‑19 responses across different countries. The GHSI, designed to assess national preparedness for health emergencies, became a key metric for evaluating how well countries managed the challenges of the pandemic. By synthesizing findings from various studies, this review explores the correlation between GHSI scores and the effectiveness of pandemic responses while identifying gaps and inconsistencies in global preparedness strategies. The findings provide insights into the strengths and limitations of the GHSI in enhancing global health security and offer recommendations for improving future pandemic preparedness frameworks.

## Research Questions

Using the scoping review methodology, this study addresses the following specific research questions:

1. How did a country’s Global Health Security Index (GHSI) score influence its capacity to respond to COVID‑19?
2. To what extent does the GHSI accurately reflect a country’s actual COVID‑19 response capacity, and what lessons can be drawn from this?

## Method

This scoping review adhered to the five‑stage framework from Arksey and O’Malley [17] and the Preferred Reporting Items for Systematic Reviews and Meta‑analyses (PRISMA) statement for scoping reviews [18] (Supplementary Table S1).

### Study selection

Data were collected from electronic databases, including EBSCO, EMBASE, PubMed, Scopus, and Web of Science. The search focused on articles published between 2020 and 2024. A comprehensive search strategy was developed to identify relevant studies using the following search string: “Global Health Index” OR “Global Health Security Index” AND (“COVID‑19” OR “COVID” OR “Corona” OR “Coronavirus” OR “Novel Coronavirus” OR “SARS‑CoV‑2”).

The full search strategy, including database‑specific search terms and filters, is provided in the supplemental materials. There is no differences existed in search terms across databases, this is explicitly stated in the supplementary materials (Table S2).

### Inclusion and exclusion criteria

The inclusion criteria encompassed peer‑reviewed original research articles published in open‑access academic journals between January 2020 and June 2024, specifically examining the Global Health Security Index (GHSI) in relation to COVID‑19. Articles published in a language other than English, review articles, commentaries, letters to the editor, editorial comments, and poster presentations were excluded.

### Data screening and extraction

**Figure 1:**
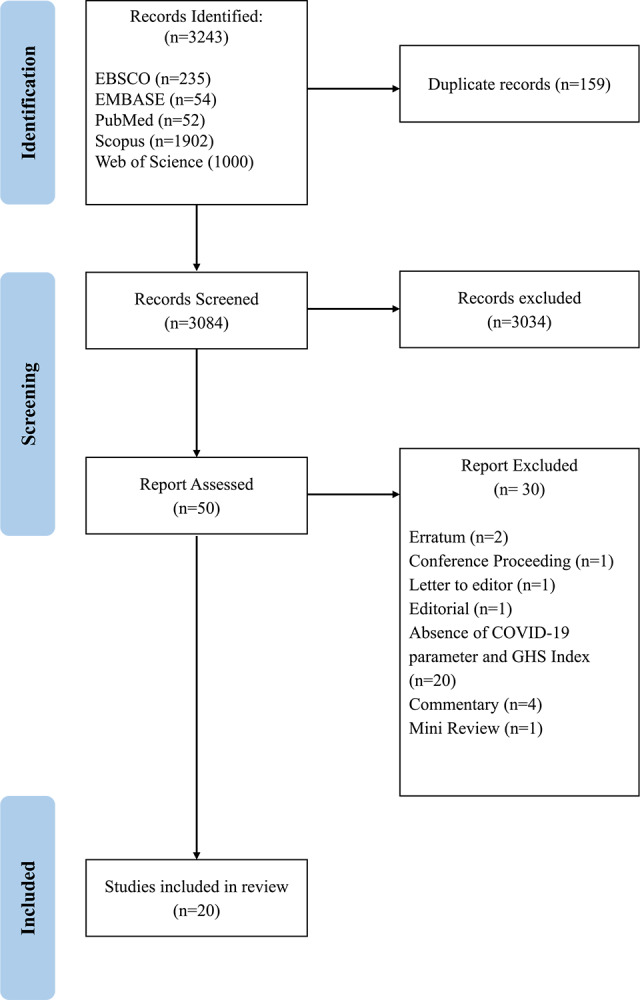
PRISMA flowchart of the study selection process.

The screening and extraction process is illustrated in Figure 1. All retrieved search results were exported from electronic databases in Research Information Systems (RIS) format and imported into EndNote 21, where duplicate entries were systematically removed. The remaining studies were then imported into Microsoft Excel 2021 [19] for title and abstract screening. Studies that did not meet the predefined inclusion criteria were excluded.

Full‑text articles were subsequently assessed for eligibility on the basis of the inclusion and exclusion criteria. Studies that lacked relevance to the Global Health Security Index (GHSI) in the context of COVID‑19 or exhibited insufficient methodological rigor were excluded.

The screening process was conducted by three independent researchers. Each researcher reviewed a portion of the retrieved articles during the initial title and abstract screening to ensure consistency and validation. During the full‑text review, all three researchers collaborated to assess eligibility. Any discrepancies in study selection were resolved through discussion, and the finalized studies were included in the data synthesis.

### Data synthesis and analysis

We performed a comprehensive descriptive analysis of the included literature, focusing on both the characteristics and thematic content of the studies. The literature was systematically categorized on the basis of two primary criteria, the specific COVID‑19 parameters used in the research and the resultant findings. These parameters were employed to provide a detailed overview of each country’s capacity to respond to the pandemic and are classified into five main groups: detection, mortality, transmission, fatality, and recovery. This classification enabled us to synthesize a broad range of research findings, offering a nuanced understanding of the role of the Global Health Security Index (GHSI) in the COVID‑19 response.

### Quality assessment

To ensure the robustness and reliability of the findings, the quality of the selected studies was assessed using the Newcastle–Ottawa Scale (NOS) [20]. This scale is specifically designed to evaluate the quality of non‑randomized studies included in meta‑analyses, with adjustments made for cross‑sectional studies (Supplementary Table S4). The NOS provides a comprehensive framework for assessing methodological rigor across three main criteria, including selection (three criteria), comparability (two criteria), and outcome assessment (three criteria). Each study was evaluated on the basis of these eight criteria. Studies scoring 7–8 points were classified as having a low risk of bias, those scoring 5–6 points were categorized as having a medium risk of bias, and studies scoring fewer than 5 points were considered to have a high risk of bias. Details of the risk of bias assessment are provided in Supplementary Table S5.

## Result

### Literature search

A total of 3,243 relevant studies were retrieved from the database search. After removing duplicates (*N* = 159), the remaining 3,084 records underwent title and abstract screening, resulting in the exclusion of 3,034 records. A full‑text review was then conducted on 50 articles, of which 30 were excluded due to the absence of COVID‑19 parameters, lack of GHSI data, or their not being original research articles. Ultimately, 20 studies were included in the final data synthesis (Figure 1). All selected articles were carefully reviewed, and their characteristics are summarized in Supplementary Table S3.

### Component of the COVID‑19 parameters

**Figure 2:**
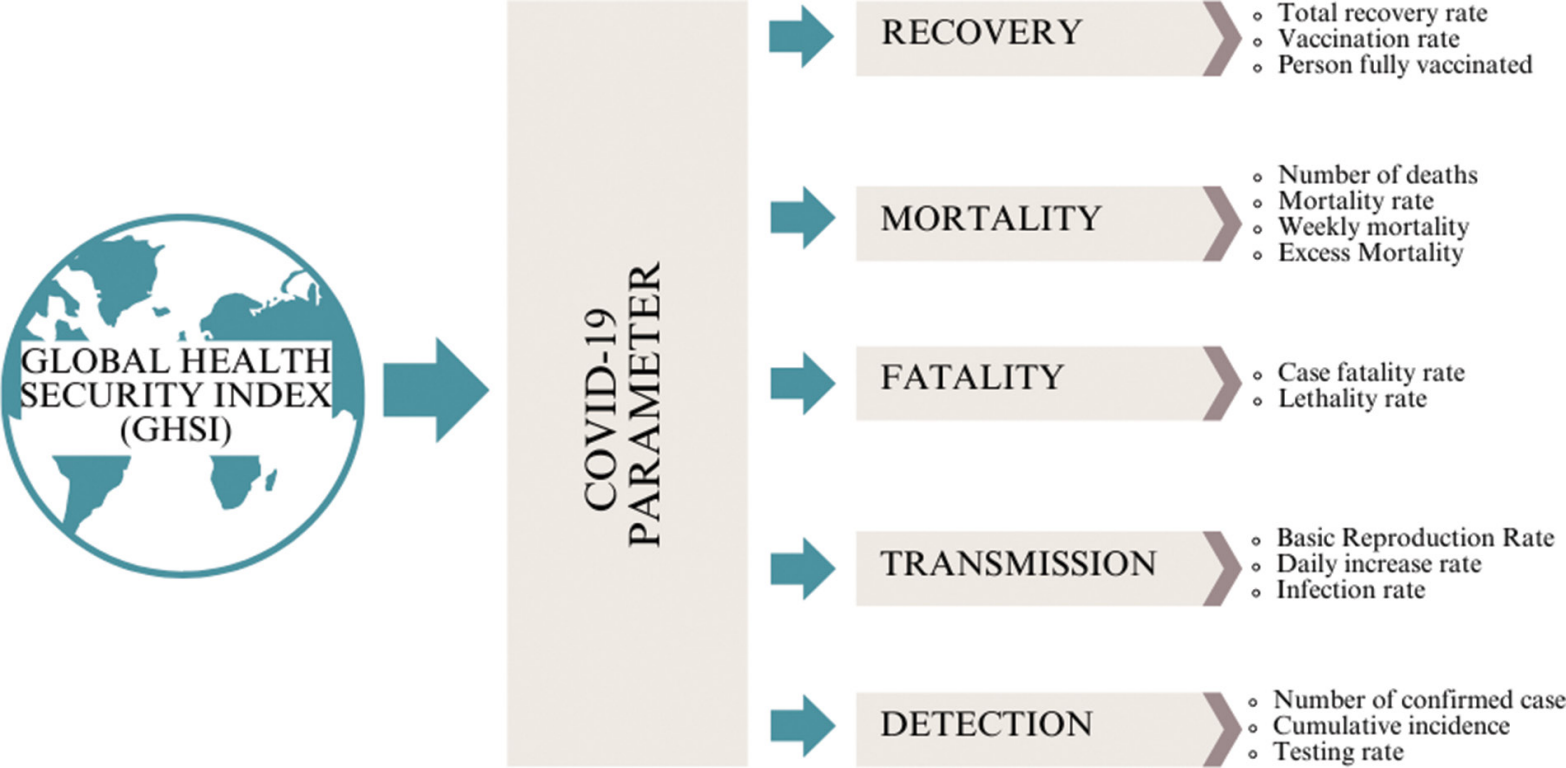
Categorization of COVID‑19 parameters used to analyze countries’ pandemic response capacities.

In this analysis, specific COVID‑19 parameters were used to provide a comprehensive overview of each country’s capacity to respond to the pandemic. These parameters are categorized into five main groups, namely detection, mortality, transmission, fatality, and recovery, as illustrated in Figure 2. Each category captures key aspects of the pandemic’s impact and evaluates the effectiveness of public health measures. Detection includes metrics such as the number of confirmed cases, cumulative incidence, and testing rates. Transmission was assessed using the basic reproduction rate (R), daily increase rate, and infection rate. Fatality was measured by the case fatality rate (CFR) and lethality rate. Mortality parameters encompassed the total number of deaths, mortality rates, weekly mortality, and excess mortality. Finally, recovery was analyzed using the total recovery rate, vaccination rate, and the number of persons fully vaccinated. These categories together provide a well‑rounded understanding of the pandemic’s dynamics and the effectiveness of interventions across countries.

### Impact of a country’s GHSI on its COVID‑19 response capacity

**Figure 3:**
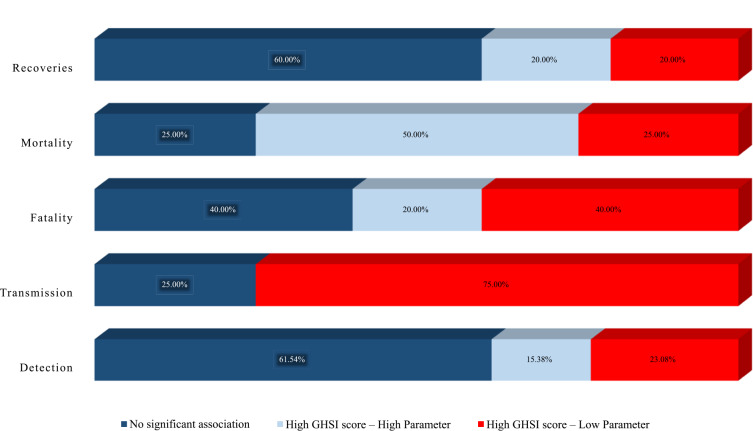
Summary of studies on the impact of GHSI scores based on COVID‑19 parameters.

Table 1 and Figure 3 summarize the relationship between GHSI scores and COVID‑19 parameters, as reported in the 20 reviewed studies.

**Table 1:** Summary of the study impact of GHSI score on COVID‑19 parameters.

COVID‑19 PARAMETER	NUMBER OF STUDIES	NO SIGNIFICANT ASSOCIATION	HIGH GHSI SCORE—HIGH PARAMETER	HIGH GHSI SCORE—LOW PARAMETER
Detection	13	[13, 15, 21–26]	[27, 28]	[29–31]
Transmission	4	[32]	—	[15, 33, 34]
Fatality	5	[29, 32]	[35, 36]	[37]
Mortality	12	[22, 26, 31]	[13, 21, 15, 24, 28, 29, 37]	[34, 37, 38]
Recovery	5	[22, 23, 31]	[28]	[13]

### Detection

Countries with higher GHSI scores were expected to have fewer confirmed cases, reflecting their enhanced ability to control virus transmission through available resources and capabilities. Of the 20 studies reviewed, 13 examined COVID‑19 detection parameters. Among these, eight studies (61.54%) [13, 15, 21–26] reported no correlation between the GHSI score and detection capacity, two studies (15.38%) [27, 28] found a positive association between higher GHSI scores and increased case confirmations, and three studies (23.08%) [29–31] reported that higher GHSI scores were linked to fewer confirmed cases.

### Transmission

Countries with higher GHSI scores were anticipated to exhibit reduced COVID‑19 transmission due to superior virus detection, prevention, and control measures. Among the 20 studies reviewed, 4 specifically addressed transmission, 3 studies (75%) [15, 33, 34] found an association between higher GHSI scores and lower transmission rates, and 1 study (25%) [32] reported no association.

### Fatality

Given their advanced healthcare facilities and resources, countries with higher GHSI scores were expected to have lower fatality rates. Among the 20 studies reviewed, 5 specifically addressed fatality. Of these, two studies (40%) [29, 32] found no association between the GHSI score and COVID‑19 fatality, one study (20%) [37] reported that higher GHSI scores were associated with lower fatality rates, and two studies (40%) [35, 36] indicated that higher GHSI scores were linked to higher fatality rates.

### Mortality

Countries with higher GHSI scores were expected to have lower COVID‑19 mortality rates due to superior healthcare capacity, workforce, and facilities. Among the 20 reviewed studies, 12 studies examined mortality. Of these, three studies (25%) [22, 26, 31] found no significant association between GHSI and mortality, seven studies (50%) [13, 21, 15, 24, 28, 29, 37] reported that higher GHSI scores were associated with higher mortality rates, and three studies (25%) [34, 37, 38] reported the opposite. Notably, one study observed that a higher GHSI was linked to higher mortality early in the pandemic but found the reverse association after 2021 [37].

### Recovery

Countries with higher GHSI scores were expected to exhibit higher recovery rates due to better healthcare infrastructure and resources. Among the 20 studies reviewed, 5 specifically addressed recovery. Of these, three studies (60%) [22, 23, 31] found no significant association between GHSI scores and recovery rates. One study (20%) [28] reported that higher GHSI scores were linked to higher recovery rates or increased vaccination rates, while the remaining study [13] reported the opposite.

### Quality appraisal

The quality appraisal of the selected studies was conducted using the Newcastle–Ottawa Scale (NOS). Among the 20 studies included, 17 (85%) were classified as having a low risk of bias, while three (15%) were categorized as having a medium risk of bias (Supplementary Table S5).

## Discussion

Our study revealed significant variation in how the Global Health Security Index (GHSI) score influenced national responses to COVID‑19. While some studies support the GHSI as a valid health security indicator correlated with stronger response capabilities, others found no significant association with actual outcomes, highlighting its limitations in predicting real‑world effectiveness.

Countries with higher Global Health Security Index (GHSI) scores were expected to report fewer confirmed cases, lower transmission and mortality rates, and higher recovery rates owing to enhanced healthcare infrastructure and early detection capabilities. However, pandemic “success” is multifaceted, shaped not only by health outcomes but also by the timing and nature of interventions, complexities that a single metric such as the GHSI cannot fully capture. Additionally, regional variations in mortality data reliability raise concerns about data completeness and comparability. Furthermore, the cross‑sectional design of many studies may limit the ability to assess the long‑term effectiveness of interventions, particularly in the evolving context of COVID‑19 [39].

Several factors should be considered when interpreting the Global Health Security Index (GHSI) as a predictor of pandemic response effectiveness. First, COVID‑19 posed an unprecedented infectious disease challenge, overwhelming the response capacities of many countries [15]. The biological characteristics of SARS‑CoV‑2, such as its ability to spread from asymptomatic individuals [40], combined with increased global connectivity [41], facilitated rapid transmission on an unprecedented scale. These factors highlight the need for preparedness metrics that account for the unique challenges posed by highly transmissible pathogens.

Second, significant disparities in access to COVID‑19 health products [42], particularly between high‑income countries (HICs) and low‑income countries (LICs), have profoundly shaped the pandemic’s trajectory. Limited initial testing capacity [15] and uneven vaccine distribution [43] exacerbated these inequalities, leading to prolonged transmission and higher infection rates in LICs. Beyond widening global health disparities, these inequities also increase the risk of new variants, which can undermine containment efforts worldwide.

Third, while the GHSI is a comprehensive metric, it heavily relies on publicly available data [44], which may overlook unreported yet effective health interventions or fail to capture the true quality of healthcare systems, particularly in lower‑income settings [23]. Fourth, the GHSI does not account for the role of political leadership in pandemic response, despite its significant influence on public health outcomes [44, 45]. The COVID‑19 pandemic underscored how policy decisions, coordination, and leadership shape crisis management [46].

For example, despite the United States’ high GHSI score, the country experienced high COVID‑19 incidence and mortality, partly due to conflicting guidance between President Trump and public health experts [47]. In contrast, South Korea [48] and New Zealand [49] demonstrated notable success through strong leadership, clear communication, and well‑coordinated responses, including the timely deployment of testing and contact tracing, resulting in relatively favorable health outcomes.

Lastly, the reliance on low‑quality data and the potential oversimplification of analyses using the GHSI may lead to misleading interpretations [35, 44]. In some cases, the observed correlation between high GHSI scores and COVID‑19 parameters such as detection, mortality, transmission, fatality, and recovery capacity may not imply a causal relationship. Instead, it may highlight limitations in data quality and the complexities of health system effectiveness. To achieve a more accurate assessment of preparedness and response, future research should incorporate longitudinal study designs and account for more nuanced factors beyond standardized metrics.

The findings from Ledesma et al. [34] suggest that countries with strong preparedness capabilities, such as effective disease prevention, detection, and robust health system infrastructure demonstrated higher data completeness and lower COVID‑19 infection and mortality rates. This supports the idea that well‑prepared nations are better equipped to track and respond to pandemics, leading to more accurate data and more effective public health interventions. Moreover, after accounting for data completeness, a negative correlation emerged between preparedness and disease burden, indicating that countries with better preparedness experienced lower infection and mortality rates.

COVID‑19 has exemplified a catastrophic infectious disease outbreak of unprecedented scale in recent decades, overwhelming the control capacities of many countries [8]. Addressing the inequality in access to health products and technology between high‑income and low‑income countries must be a priority in future pandemic treaties [42]. The significant disparities in access to COVID‑19‑related health products and technologies not only deepen global health inequities but also risk prolonging the pandemic, increasing the likelihood of new variants, and undermining global containment efforts [50].

The uneven distribution of vaccines [43], treatments, and diagnostics [42] has slowed infection rate declines in low‑income countries, increasing the risk of sustained transmission and the emergence of new variants that could jeopardize global containment efforts. Developed nations typically achieve higher GHSI scores due to their greater capacity and resources, whereas lower GHSI scores in developing countries reflect systemic resource limitations. These disparities exacerbate existing social inequalities, further marginalizing vulnerable populations and weakening global solidarity. To address these challenges, future preparedness frameworks should incorporate measures that evaluate equitable access to healthcare resources.

Our study emphasizes that the GHSI did not accurately reflect a country’s capacity to respond to the COVID‑19 pandemic. However, this does not undermine the value of the GHSI, which is intended to identify gaps that countries must address [44]. It remains a valuable tool for identifying essential capacities and capabilities that are necessary, though not sufficient, for an effective pandemic response [39]. Rather, this finding underscores the complex and unpredictable nature of pandemics, which are shaped by factors that extend beyond the current GHSI metrics.

A key recommendation is for the expert panel overseeing the GHSI to regularly reassess its criteria, integrating additional variables such as leadership quality, governance, public health infrastructure, and socio‑cultural factors. These elements have proven essential in shaping effective pandemic responses. Including such factors in the GHSI would enhance its ability to reflect a country’s true preparedness and response capabilities, providing a more comprehensive tool for global health security. By evolving to incorporate a broader range of indicators, the GHSI can better guide countries in strengthening their health security frameworks, ultimately leading to improved outcomes during future public health crises.

### Limitation

This scoping review aimed to offer a comprehensive overview of the scientific literature regarding the impact of the Global Health Security Index (GHSI) on countries’ responses to the COVID‑19 pandemic. However, several limitations may affect the scope and completeness of the review. First, the review focused solely on English‑language publications from 2020 to 2024, which could exclude significant studies published in other languages. This linguistic limitation may have omitted valuable international research and diverse perspectives that could provide deeper insights into the GHSI’s impact. Second, the exclusion of gray literature, such as reports, theses, conference papers, and government documents may have resulted in an incomplete representation of available data. Gray literature is particularly important in dynamic situations such as a pandemic, where findings may not immediately appear in peer‑reviewed journals. These limitations suggest that, while the review offers valuable insights, its findings may be partial. Future research would benefit from broader inclusion criteria to ensure a more comprehensive assessment of the literature and a fuller understanding of the topic.

## Conclusion

Our study emphasizes that the Global Health Security Index (GHSI) does not fully capture a country’s ability to respond effectively to the COVID‑19 pandemic. However, this does not undermine the GHSI’s value. Instead, it highlights the need for its continuous evolution. The GHSI remains a vital tool for identifying critical gaps in the capacities and capabilities required for an effective pandemic response. To improve its accuracy and relevance, the index should incorporate a wider range of variables, such as political leadership, governance, public health infrastructure, and socio‑cultural factors, all of which play pivotal roles in managing public health crises.

Regular reassessment and refinement of the GHSI criteria would not only enhance its predictive power but also support countries in identifying and addressing deficiencies in their health security systems. By embracing this more comprehensive approach, the GHSI can evolve into a more robust and reliable instrument for global health preparedness, ultimately strengthening resilience against future pandemics.

## Availability Data and Materials

Since this study is a scoping review, all data and materials utilized are sourced from published literature.

## Abbreviations

COVID‑19: Coronavirus disease 2019

GHS: Global Health Security

GHSI: Global Health Security Index

SARS: Severe Acute Respiratory Syndrome

## Additional Files

The additional file for this article can be found as follows:

Supplementary Table S1. Preferred Reporting Items for Systematic Reviews and Meta‑analyses Extension for Scoping Reviews (PRISMA‑ScR) checklist.

Supplementary Table S2. Study search strategy.

Supplementary Table S3. Summary of the characteristics of the final studies.

Supplementary Table S4. The Newcastle–Ottawa Scale (NOS) adjusted for cross‑sectional studies.

Supplementary Table S5. The Newcastle–Ottawa Scale (NOS) assessment result.
