# Supplementary material for: The Global Health Security Index and Its Role in Shaping National COVID‑19 Response Capacities: A Scoping Review
Source: Ann Glob Health. 2025 Mar 14;91(1):15. doi: 10.5334/aogh.4625 (PMC11908420; doi:10.5334/aogh.4625)
Supplement: Supplementary Table S2. — Study search strategy. [file agh-91-1-4625-s2.pdf]

Table S2. Study Search Strategy

Databases Searched

The literature search was conducted in the following electronic databases:

- EBSCO
- EMBASE
- PubMed
- Scopus
- Web of Science

Search Terms Used

The same search string was applied across all databases:

“Global Health Index” OR “Global Health Security Index” AND (“COVID-19” OR “COVID” OR “Corona” OR “Coronavirus” OR “Novel Coronavirus” OR “SARS-CoV-2”)

Search Date

The search was conducted on 1ST July 2024.

Filters and Limits Applied

- **Publication Date:** January 2020 – June 2024
- **Language:** English only
- **Study Type:** Peer-reviewed journal articles

Search Results per Database

Database	Number of Articles Retrieved
EBSCO	235
EMBASE	54
PubMed	52
Scopus	1902
Web of Science	1000

Note: No differences in search terms were observed across databases.
