# Supplementary material for: The Global Health Security Index and Its Role in Shaping National COVID‑19 Response Capacities: A Scoping Review
Source: Ann Glob Health. 2025 Mar 14;91(1):15. doi: 10.5334/aogh.4625 (PMC11908420; doi:10.5334/aogh.4625)
Supplement: Supplementary Table S3. — Summary of the characteristics of the final studies. [file agh-91-1-4625-s3.pdf]

Table S3. Summary of the Characteristics of the Final Studies

No	Author	Year	GHSI (Year)	Research Design	COVID-19 Parameter	Countries Analyzed	Key Findings	Practical Implications
1	Abbey, et al.	2020	2019	Cross-sectional	• COVID-19 Cases<br>• COVID-19 deaths,<br>• COVID-19 recoveries,<br>• COVID-19 number of tests	36	• The GHS Index does not accurately predict pandemic responses among OECD countries.	• The GHS Index may underestimate the preparedness, reassessment, and leadership inclusion required.<br>• Previous health threat responses should inform future GHS Index reports.
2	Haider, eat al.	2020	2019	Cross-Sectional	• COVID-19 Death per Million<br>• First case Detection of COVID-19	168	• GHSI scores are not correlated with COVID-19 detection or mortality rates<br>• Mortality rates in countries are linked to population age, not GHSI scores	• The GHSI did not effectively predict COVID-19 outcomes.<br>• Countries with higher importation risk detected cases earlier.<br>• Investing in long-term health infrastructure is essential for pandemic preparedness.
3	Amadu, et al.	2021	2019	Cross-Sectional	• COVID-19 Cases per million<br>• COVID-19 Recoveries per Million<br>• COVID-19 Death per Million	54	• There are strong correlations between COVID-19 cases, deaths, and recoveries with various factors.<br>• Spatial representations of COVID-19 data across African regions are provided.<br>• High death rates are observed in the least prepared countries, in contrast to their recovery rates	• Countries with high GHSI metrics manage COVID-19 cases more effectively.<br>• National governments should implement interventions based on GHSI benchmarks.
4	Costa, et al.	2021	2019	Cross-Sectional	• Cumulative incidence per 100.000<br>• Cumulative mortality per 100,000<br>• The lethality per 1,000 cases,<br>• The testing rate<br>• Excess mortality attributed to COVID-19	50	• Countries with high GHSI scores experienced more cases, deaths, and excess mortality.<br>• The lack of vaccines contributed to the spread of COVID-19 in 2020.<br>• The GHSI correlates with wealth and an older population.	• The GHSI needs to incorporate political and institutional variables for improvement.<br>• The role of governments in enforcing social distancing impacts support for the health sector.<br>• High-income countries struggled to protect against COVID-19 due to demographic factors.<br>• Societal choices influenced COVID-19 outcomes in countries with low GHSI scores.

No	Author	Year	GHSI (Year)	Research Design	COVID-19 Parameter	Countries Analyzed	Key Findings	Practical Implications
5	Ji, et al.	2021	2019	Cross-Sectional	• Total cases per million<br>• Total Deaths per million<br>• Daily increase rate	142	• The GHS Index was inversely correlated with COVID-19 mobility and mortality.<br>• Lockdown measures significantly reduced the rate of increase in COVID-19 cases.	• The GHS Index correlates with COVID-19 mobility and mortality rates.<br>• The GHSI has limited value in assessing global pandemic responses but is useful for local epidemics.<br>• Implementing lockdown measures can reduce the rate of COVID-19 cases.
6	Khalifa, et al.	2021	2019	Cross-Sectional	• Total number of COVID-19 cases / million persons<br>• Cumulative COVID-19 vaccines / hundred<br>• The percentage of the population fully vaccinated (i.e., who received both doses of a two-dose vaccine, or a single dose of a one-dose vaccine) at 2 months after initiation of the vaccination program in each country.	33	• The correlation between the GHS Index and vaccination rates was insignificant.	• There is a lack of correlation between the GHS Index and COVID-19 vaccine rollout.<br>• Political and cultural differences, as well as vaccine hesitancy, should be considered in preparedness frameworks
7	Leichtweis, et al.	2021	2019	Cross-Sectional	Basic Reproduction Number (R0)	52	• The GHS Index had the greatest explanatory power in controlling COVID-19<br>• Countries with high GHS scores are less influenced by climate variables<br>• The basic reproduction number (R_0) is lower in countries with advanced health systems and political stability.	• Climate variables and the GHS Index impact the spread of COVID-19 in different ways.<br>• Temperature, solar radiation, and the GHS Index influence virus transmission rates.
8	Duong, et al.	2022	2019	Cross-Sectional	COVID-19 cases per million	195	• Higher GHSI scores are linked to fewer COVID-19 cases.<br>• GHSI sub-scales show a significant association with COVID-19 outcomes.	• The GHSI can assist in assessing pandemic preparedness<br>• Future indices should account for outbreak stages and sub-national scores
9	Goldschmidt	2022	2019	Cross-Sectional	• COVID-19 case fatality rates<br>• COVID-19 per capita death rates<br>• Excess mortality	20	• The GHSI score initially correlated with higher COVID death rates.<br>• By July 2021, the GHSI score correlated with lower COVID death rates.	• Evaluating pandemic preparedness and performance based on achievable outcomes is crucial.

No	Author	Year	GHSI (Year)	Research Design	COVID-19 Parameter	Countries Analyzed	Key Findings	Practical Implications
							• The GHSI score was more strongly correlated with excess mortality.<br>• Per capita GDP was a good predictor of excess mortality.	• Premature evaluation of pandemic response management can be undermined by subsequent events.<br>• The effectiveness of countries' pandemic response management can only be fully assessed post-pandemic.
10	Kumru, et al.	2022	2019	Cross-Sectional	• Confirmed cases of COVID-19 per million population<br>• Confirmed deaths from COVID-19 per million population	92	• Canonical correlation analysis revealed significant associations between COVID-19 indicators and various variables.<br>• Strong correlations were observed between COVID-19 cases, deaths, and independent variables.	• Socioeconomic factors significantly impact COVID-19 cases and deaths.<br>• Countries' performances vary, with some being more successful in managing the pandemic.
11	Legese, et al	2022	2019	Cross-Sectional	COVID-19 cases per million	166	• Civil liberties, health security, median age, and population size impact the spread of COVID-19.<br>• Tests per million, population size, and the civil liberties index affect the spread of COVID-19.<br>• The Health Security Index negatively affects the number of confirmed COVID-19 cases.	• The government should carefully align civil rights with public health protection.<br>• Measures to restrict civil liberties must be proportionate.
12	Markovic, et al.	2022	2019	Cross-Sectional	Case Fatality Ratio (CFR)	85	• GHSI categories were positively correlated with measures of COVID-19 severity.<br>• Age and epidemic onset are significant predictors of COVID-19 severity.<br>• Excess deaths are linked to COVID-19 counts and demographic variables.<br>• Developed countries have fewer unexplained COVID-19 deaths.<br>• High GHSI values are not linked to an unsatisfactory pandemic response.	• High GHSI values do not correlate with pandemic response effectiveness or severity.<br>• Analysis of excess deaths is more reliable than official COVID-19 figures.<br>• GHSI values do not predict unsatisfactory pandemic responses or high mortality.
13	Maruta and Moyo	2022	2019	Cross-Sectional	COVID-19 cases per million	42	• Countries with higher preparedness had fewer COVID-19 cases per million people.	• Highlighted need for pandemic preparedness in African countries.<br>• Emphasized importance of assessing critical areas for epidemic handling.

No	Author	Year	GHSI (Year)	Research Design	COVID-19 Parameter	Countries Analyzed	Key Findings	Practical Implications
							• Laboratory systems and real-time surveillance impacted the number of COVID-19 cases	
14	Nazari, et al.	2022	2019	Cross-Sectional	• Weekly standardized number of COVID-19 cases<br>• Weekly standardized number of COVID-19 deaths.	54	• GHSI scores did not align with COVID-19 incidence or preparedness.<br>• Incidence rates varied among countries based on their GHSI levels.	• GHSI may not fully predict COVID-19 outcomes accurately.<br>• The results can guide countries in improving their COVID-19 control strategies.<br>• The study suggests modifying or developing new health security indices.
15	Tan, et al.	2022	2019	Cross-Sectional	• Case Fatality Ratio (CFR)	4	• Every 1-point increase in the Global Health Security (GHS) index score may increase the 3 weeks lagging CFR by 0.12%	• The regression results showed higher GHS index was a factor that increased CFR, but the GHS index cannot perfectly assess a country's ability to respond to a global pandemic.
16	Șoitu, et al.	2022	2021	Cross-Sectional	• Confirmed COVID-19 cases<br>• COVID-19 Death Rate<br>• Proportion of Population Fully Vaccinated	27	• The EU-27 has limited capabilities in health security indexing.	• Link medical, social, and economic variables to political regimes during the pandemic.<br>• Emphasize the importance of democracy and resilience in healthcare systems.<br>• Predictors of health security include social progress, human development, and democracy.<br>• Develop a theoretical model to assess the political impact on quality of life.
17	Alhassan, et al.	2023	2021	Cross-Sectional	• Cumulative COVID-19 cases (numeric)<br>• Cumulative COVID-19 deaths (numeric)<br>• Persons fully vaccinated (numeric)<br>• Persons fully vaccinated per 100 population (numeric).	195	• High GHSI scores did not reduce COVID-19 cases as expected.<br>• The GHSI is not a reliable indicator for predicting COVID-19 response.	• The GHSI may not predict COVID-19 response effectively, suggesting the need for methodology revisions.<br>• GHS and UHC must be balanced to create resilient health systems.

No	Author	Year	GHSI (Year)	Research Design	COVID-19 Parameter	Countries Analyzed	Key Findings	Practical Implications
18	Biadgilign, et al	2023	2019	Cross-Sectional	• COVID-19 case fatality (per 10.000)<br>• COVID-19 infection rates (per 10.000)	54	• GHS directly affects SARS-CoV-2 infection and case-fatality rates in Africa.<br>• GHS does not protect against COVID-19-related case fatality rates.<br>• Structural Equation Modeling results show significant relationships between variables in COVID-19 outcomes.	• UHC and GHS impact COVID-19 rates in Africa.<br>• Strong UHC can reduce COVID-19 infection rates.<br>• Addressing poverty and promoting development are crucial for better healthcare outcomes.
19	Ledesma, et al	2023	2021	Cross-Sectional	Total COVID-19 excess mortality	22	• The GHS Index is negatively associated with excess COVID-19 case mortality rates.<br>• Sensitivity analysis showed no significant relationships between the GHS Index and case mortality rates.	• Greater health security capacities are linked to lower COVID-19 mortality rates.<br>• Pandemic preparedness measures can mitigate the impact of infectious diseases.<br>• Improved pandemic outcome measures are needed for an unbiased understanding of preparedness.
20	Ledesma, et al.	2024	2021	Cross-Sectional	• SARS-CoV-2 infection completion rates<br>• SARS-CoV-2 death completion rate<br>• SARS-CoV-2 infection rate at 100 days<br>• Age-standardized COVID-19 death rates	195	• The analysis indicated that every 10% increase in the GHS Index was associated with a 14.9% increase in infection data completeness and a 10.6% increase in death data completeness during the observed period.<br>• The study also found that higher GHS Index scores were linked to lower infection and age-standardized COVID-19 death rates when accounting for differences in data completeness.	• The results supported the idea that well-prepared countries had better surveillance and reporting systems, thus enabling more accurate tracking and response to the pandemic

*CFR: Case Fatality Rate; COVID-19: Corona Virus Disease 2019; GHSI: Global Health Security Index; OECD: The Organization for Economic and Cooperation Development.
