# Supplementary material for: The Global Health Security Index and Its Role in Shaping National COVID‑19 Response Capacities: A Scoping Review
Source: Ann Glob Health. 2025 Mar 14;91(1):15. doi: 10.5334/aogh.4625 (PMC11908420; doi:10.5334/aogh.4625)
Supplement: Supplementary Table S4. — The Newcastle–Ottawa Scale (NOS) adjusted for cross‑sectional studies. [file agh-91-1-4625-s4.pdf]

Table S4. The Newcastle-Ottawa Scale (NOS) - Adjusted for Cross Sectional

**Selection**	
Study Objective	1 Point is awarded if the study objective is clearly stated in the paper;
Sample Size	1 point is awarded if the sample is either truly representative of the target population (e.g., through all subjects or random sampling) or somewhat representative (e.g., through non-random sampling).
Inclusion and Exclusion	1 point is awarded if the inclusion and exclusion criteria for subjects are clearly defined.
**Comparison**	
Data Source	1 point is awarded if the data is sourced from reliable references or is provided directly within the paper
Data Collection	1 point is awarded if the data collection method and period are clearly stated in the paper.
**Outcomes**	
Confounding Identification	1 point is awarded if potential confounders are identified and clearly stated.
Confounding Adjustment	1 point is awarded if a method to address potential confounders is implemented.
Confounding Adjustment	1 point is awarded if the statistical test used to analyze the data is clearly described, appropriate for the study, and if the measurement of the association is presented.

Categorization:

- 7 – 8 points : Low risk bias
- 5 – 6 points : Medium risk bias
- < 5 points : High risk bias
