# Supplementary material for: The Global Health Security Index and Its Role in Shaping National COVID‑19 Response Capacities: A Scoping Review
Source: Ann Glob Health. 2025 Mar 14;91(1):15. doi: 10.5334/aogh.4625 (PMC11908420; doi:10.5334/aogh.4625)
Supplement: Supplementary Table S5. — The Newcastle–Ottawa Scale (NOS) assessment result. [file agh-91-1-4625-s5.pdf]

Table S5. The Newcastle-Ottawa Scale (NOS) Assessment Result

No	Author	Year	GHSI (Year)	Selection			Comparison		Outcomes			Score	Criteria
				Study Objective	Sample size	Inclusion and Exclusion	Data source	Data collection	Confounding Identification	Confounding Adjustment	Statistical Analysis		
1	Abbey, et al.	2020	2019	1	1	1	1	1	1	1	1	8	LOW RISK
2	Haider, eat al.	2020	2019	1	1	1	1	1	1	1	1	8	LOW RISK
3	Amadu, et al.	2021	2019	1	1	0	1	1	1	1	1	7	LOW RISK
4	Costa, et al.	2021	2019	1	1	0	1	1	1	1	1	7	LOW RISK
5	Ji, et al.	2021	2019	1	1	1	1	1	1	1	1	8	LOW RISK
6	Khalifa, et al.	2021	2019	1	1	1	1	1	1	0	1	7	LOW RISK
7	Leichtweis, et al.	2021	2019	1	1	0	1	1	1	1	1	7	LOW RISK
8	Duong, et al.	2022	2019	1	1	1	1	1	1	1	1	8	LOW RISK
9	Goldschmidt	2022	2019	1	1	0	1	1	1	0	1	6	MEDIUM RISK
10	Kumru, et al.	2022	2019	1	1	0	1	1	1	1	1	7	LOW RISK
11	Legese, et al	2022	2019	1	1	0	1	1	1	0	1	6	MEDIUM RISK
12	Markovic, et al.	2022	2019	1	1	1	1	1	1	1	1	8	LOW RISK

No	Author	Year	GHSI (Year)	Selection			Comparison		Outcomes			Score	Criteria
				Study Objective	Sample size	Inclusion and Exclusion	Data source	Data collection	Confounding Identification	Confounding Adjustment	Statistical Analysis		
13	Maruta and Moyo	2022	2019	1	1	1	1	1	1	1	1	8	LOW RISK
14	Nazari, et al.	2022	2019	1	1	1	1	1	1	1	1	8	LOW RISK
15	Tan, et al.	2022	2019	1	1	1	1	1	1	1	1	8	LOW RISK
16	Șoitu, et al.	2022	2021	1	0	0	1	1	1	1	1	6	MEDIUM RISK
17	Alhassan, et al.	2023	2021	1	1	1	1	1	1	1	1	8	LOW RISK
18	Biadgilign, et al	2023	2019	1	1	0	1	1	1	1	1	7	LOW RISK
19	Ledesma, et al	2023	2021	1	1	0	1	1	1	1	1	7	LOW RISK
20	Ledesma, et al	2024	2021	1	1	0	1	1	1	1	1	7	LOW RISK
